# *Centella asiatica* extract‐SiO_2_ nanocomposite: More than a drug‐delivery system for skin protection from oxidative damage

**DOI:** 10.1002/jbm.a.37462

**Published:** 2022-10-24

**Authors:** Federico Ebau, Alessandra Scano, Maria Letizia Manca, Maria Manconi, Valentina Cabras, Martina Pilloni, Guido Ennas

**Affiliations:** ^1^ Chemical and Geological Sciences Department, University of Cagliari, Research Unit of the National Consortium of Materials Science and Technology (INSTM) Cittadella Universitaria di Monserrato Monserrato Italy; ^2^ Life and Environment Science Department, Section of Drug Sciences, CNBS University of Cagliari Cagliari Italy

**Keywords:** antioxidant activity, ball milling, biomaterial, *Centella asiatica*, fumed silica, nanocomposites, oxidative damage, skin protection

## Abstract

An innovative nanotechnology‐based approach was used for the preparation of *Centella asiatica* (*C. asiatica*) extract‐SiO_2_ nanocomposites, specifically tailored for skin protection from oxidative damage. Different amounts of *C. asiatica* glycolic extract (1.0, 3.0, 5.0, and 10.0 wt %) and fumed silica were used to prepare the nanocomposites by means of ball milling method. The influence of both composition of the starting mixture and milling time on the final products was evaluated by different techniques such as X‐ray powder diffraction, scanning electron microscopy, infrared spectroscopy, thermogravimetric analysis, and nitrogen sorption analysis. Results confirmed the integrity of the natural extract after the milling process, and its successful loading in the silica matrix. No cytotoxicity was observed for the obtained nanocomposites, which showed high in‐vitro ability to scavenge 2,2‐diphenyl‐1‐picrylhydrazyl and to protect human keratinocytes from damages induced with hydrogen peroxide.

## Graphical abstract

*Centella asiatica* extract‐SiO_2_ nanocomposites, specifically tailored for skin protection from oxidative damage were prepared by an innovative nanotechnology‐based approach.

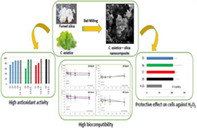

## 1 INTRODUCTION

Recently, great attention has been devoted to the use of medicinal plants in the therapeutic treatment of skin diseases, mainly because of the antioxidant and anti‐inflammatory properties of their bioactives. On this regard, *Centella asiatica* (*C. asiatica*) is of particular interest.^1^, ^2^, ^3^, ^4^, ^5^, ^6^, ^7^ It commonly grows in many parts of the world, including Asia where has long been used in traditional medicine due to its therapeutic properties for tissue regeneration,^8^ cell migration,^9^ and wound repairing.^10^, ^11^ The latter is related to its ability to promote fibroblast proliferation and collagen synthesis mainly due to saponin‐containing triterpene acids and their sugar esters such as asiaticoside, asiatic and madecassic acid, as well as other phytochemical constituents such as flavonoids, sesquiterpenes, plant sterols, eugenol derivatives and caffeoylquinic acids.^12^, ^13^, ^14^ Unfortunately, the in‐vivo efficacy of its main components after skin application is limited by their low bioavailability especially when formulated in topical ointments.^15^

We recently developed a one‐pot‐production method loading silica matrix with active substances using ball milling, which allows the retention of the properties of the bioactive after the milling process along with their metabolic composition.^16^, ^17^, ^18^ In a previous study, *Vitis Vinifera* ethanolic extract was delivered in the silica matrix and the obtained nanocomposite was able to improve its antioxidant activity.^17^ According to these promising results, in this paper, we present a novel nanocomposite based on *C. asiatica* glycolic extract and fumed SiO_2_, which was prepared as new strategy to treat skin damages linked to oxidative stress. In this new biomaterial concept, *C. asiatica* can scavenge free radicals at the skin lesion site and can block an abnormal growth of collagen‐producing keloid cells, together with antimicrobial and anti‐inflammatory effects.^19^, ^20^ On the other side, the silica matrix can act as both drug delivery system and promoter of wound healing. Indeed, our previous studies already showed the controlled drug release capabilities of the silica matrix^18^ while several reports suggest that silica can affect tissue repair by promoting cytokine generation for collagen synthesis (scarring), and the generation of new blood vessels (angiogenesis).^21^, ^22^ Moreover, our new strategy benefits of the high availability of silica in nature, cost‐effective and easy to scale‐up production of the nanocomposites.^23^, ^24^ Along with the advantages described before, the incorporation of natural products in the silica‐based delivery system can protect the natural extract from physical and chemical degradation, therefore enhancing its bioavailability and pharmacological activity.^17^, ^18^

## 2 MATERIALS AND METHODS

### 2.1 Nanocomposite preparation

C. asiatica glycolic extract (2:1 E/D ratio) was purchased from Galeno srl (Comeana, Italy). Fumed Silica (99.8%) was purchased from Sigma Aldrich. All the reagents were used as received without further purification.

In 1 g of fumed silica and variable amounts of *C. asiatica* glycolic extract were sealed in a 60 ml agate vial with 22.68 g of agate balls. Different compositions of the starting mixture were selected to obtain final nanocomposites with a dry extract content of *C. asiatica* equal to 1.0, 3.0, 5.0, and 10.0 wt % (Table 1). Ball milling was performed in a planetary mill apparatus (Fritsch GmbH, Pulverisette 5) at 100 rpm, alternating milling and rest periods at 5 min intervals to prevent an excessive overheating of the vial. All the samples were dried at room temperature for 48 h after the milling process.

**TABLE 1:** Composition of samples, milling time and textural properties

Sample	(*Centella asiatica*) (wt %)	Milling time (min)	SSA (m^2^/g)^a^	Dp (nm)	Vp (cm^3^/g)^b^
SiO_2_	—	—	203	2.8	0.93
S1	1	10	168	2.6	1.33
S2	1	30	168	2.6	1.24
S3	1	60	109	2.5	0.85
S4	1	120	117	2.6	0.78
S5	3	60	24	2.3	0.35
S6	3	120	69	2.4	0.87
S7	5	60	—	—	—
S8	5	120	—	—	—
S9	10	30	—	—	—

^a^ Calculated by the BET method.

^b^ Total pore volume of pores less than 2278.78 Å width at p/p_0_ = 0.99.

### 2.2 Nanocomposite characterization

X‐ray powder diffraction (XRPD) data were collected using CuKα radiation on a Seifert XRD 3000 TT diffractometer in the Bragg–Brentano geometry with a step size of 0.05° 2*ϑ* degrees, in the angle range 5° < 2*ϑ* < 80°. An appropriate number of counts for each step was collected to improve the signal/noise ratio.

The morphology of the nanocomposites was studied by scanning electron microscope (SEM). Samples were fixed on a brass stub with double‐sided carbon coated with gold (Blazers SCD 004 sputter coater for 2 min) and observed under an excitation voltage of 10 kV using a SEM S‐4100, HITACHI.

Fourier‐transform infrared spectroscopy (FTIR) analysis of the starting mixture and of the nanocomposites was carried out with a Bruker Tensor 27 spectrophotometer, equipped with a diamond‐ATR accessory and a DTGS detector. The 128 scans at a resolution of 2 cm^−1^, were performed at a wave number ranging from 4000 to 400 cm^−1^.

Nitrogen sorption isotherms were obtained on an ASAP2020 apparatus operating at 77 K. In order to avoid the decomposition of the organic components in the extract during the outgassing protocol, 80°C was selected as the temperature of the outgassing step. It was chosen considering the thermal stability of the natural extract, which was observed by thermogravimetric (TG) and FTIR analysis (data not reported). The specific surface area (SSA) was calculated by Brunauer–Emmett–Teller (BET) equation.^25^, ^26^ For the total pore volume (Vp), the single point adsorption at p/p_0_ = 0.99 was considered.

TGA was carried out at atmospheric pressure using a Perkin Elmer instrument model TGA7, working under Ar flow (40 ml min^−1^), in the temperature range of 30–800°C with a heating rate of 10°C min^−1^. The instrument was calibrated with Curie points of Alumel, Nickel, Perkalloy and Iron standard samples (accuracy: ±2°C).

### 2.3 DPPH radical scavenging assay

The 2,2‐diphenyl‐1‐picrylhydrazyl (DPPH) and methanol (HPLC Plus, ≥99.9%) were purchased from Sigma Aldrich.

The ability of samples to scavenge DPPH radicals was measured adding 40 μl of the *C. asiatica* extract contained in each nanocomposite to 960 μl of DPPH methanolic solutions. DPPH methanolic solution at the same dilution was also used as control. Absorbance was measured at 517 nm after storage at room temperature for 30 min in the dark, using a UV instrument Perkin Elmer mod. Lambda 25 (Monza, Italy). Experiments were performed in triplicate. The free radical scavenging activity, expressed as percentage of antioxidant activity (AA%), was calculated according to the following formula^27^:

$$\mathrm{AA}\%=\left[\left(A_{\text{DPPH}}-A_{\text{sample}}\right)/A_{\text{DPPH}}\right]*100,$$

where A is the absorbance.

The obtained results were compared with the activity of the pure *C. asiatica* glycolic extract at the same concentration.

### 2.4 In‐vitro evaluation of the nanocomposite biocompatibility

Cell medium, fetal bovine serum, phosphate buffer solution (PBS), penicillin, streptomycin and all the other reagents for cell studies were purchased from Thermo Fisher Scientific Inc. (Waltham, MA, US). Fresh bidistillate water was also used.

Biocompatibility evaluation was carried out on the selected as more representative samples (S2, S6, S7 and S9 samples). Human keratinocytes (HaCaT) were chosen for in‐vitro experiments. Cells, at passages 4–5, incubated in 5% CO_2_ at 37°C, were cultured by using RPMI1640, supplemented with fetal bovine serum, penicillin/streptomycin and fungizone as culture medium. Cell viability was measured by means of the 3‐[4,5‐dimethylthiazol‐2‐yl]‐3,5 diphenyl tetrazolium bromide (MTT) colorimetric test.^28^ Briefly, cells (7.5 × 10^3^/well) were seeded into 96‐well plates, cultured for 24 h, and then treated for 24 and 48 h with the samples. After 24 and 48 h of treatment, the MTT solution (100 μl, 0.5 mg/ml final concentration) was added to each well, removed after 3 h, and replaced with dimethyl sulfoxide (100 μl). The absorbance of the solubilized dye was read at 570 nm by using a microplate reader (Multiskan EX, Thermo Fisher Scientific Inc., Waltham, MA, US). The results are shown as percentage of live cells in comparison with untreated control cells (100% cell viability).

### 2.5 Evaluation of the nanocomposite protective effect against cell damages

HaCaT were seeded into 96‐well plates and incubated at 37°C in 5% CO_2_ for 24 h. The cells were stressed with hydrogen peroxide (25 μl, 1:40000 dilution), and straightaway incubated for 4 h with 25 μl of the extracts in dispersion or loaded in nanocomposites (2 μg/ml of extract, final concentration). The cells not stressed with hydrogen peroxide and untreated with the formulations (100% of viability) were used as a positive control. The cells stressed with hydrogen peroxide and untreated with the formulations were used as a negative control. After 4 h, the cells were washed with PBS, and the MTT assay was performed as described above, to evaluate the viability of cells after the treatment.^29^

## 3 RESULTS AND DISCUSSION

### 3.1 Structural, morphological, textural, and thermal characterization

**FIGURE 1:**
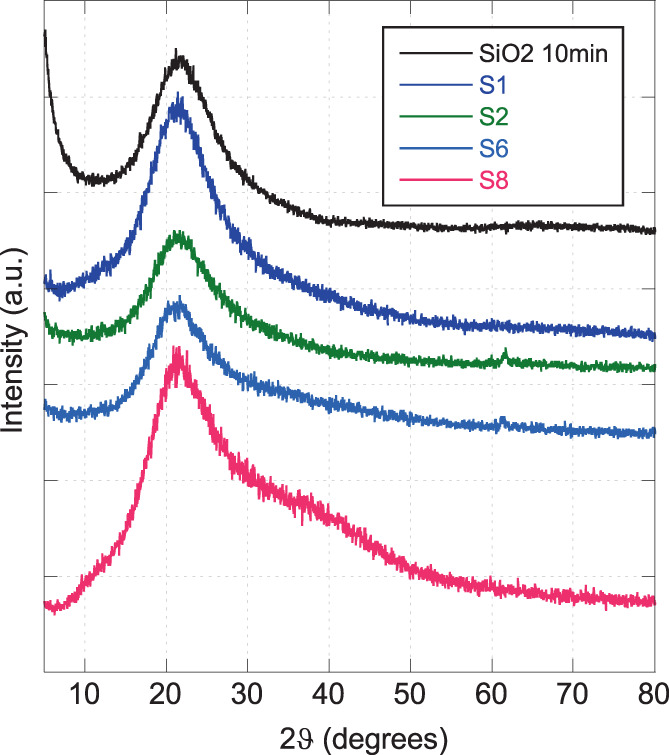
X‐ray powder diffraction patterns of the milled fumed silica and of the most representative nanocomposites (S1, S2, S6, and S8 samples)

Several extract‐SiO_2_ nanocomposites were prepared using 1.0, 3.0, 5.0, and 10.0 wt % of dry extract and different milling times (10, 30, 60, and 120 min). The milling and extract concentration effect on the structural properties of nanocomposites were monitored by XRPD analyses performed on small portions of the samples. Figure 1 reports the most representative XRPD patterns. Typical halo of the amorphous silica is visible in the range between 15 and 30° 2ϑ degrees in the patterns of all the samples. Some contributions due to the *C. asiatica* extract are visible in the nanocomposites S4 and S6 (weak peak at 61° 2*ϑ*) and in the S8 sample (bump in the range 30–50° 2*ϑ*). Such contributions are not visible in the milled pure SiO_2_. In the pattern of samples prepared with the same amount of extract but different milling time, an enlargement of the SiO_2_ haloes can be observed (e.g. S1 and S4 samples, Figure 1), due to the increase of the system disorder related to an increment of the milling time.^18^, ^19^, ^24^

**FIGURE 2:**
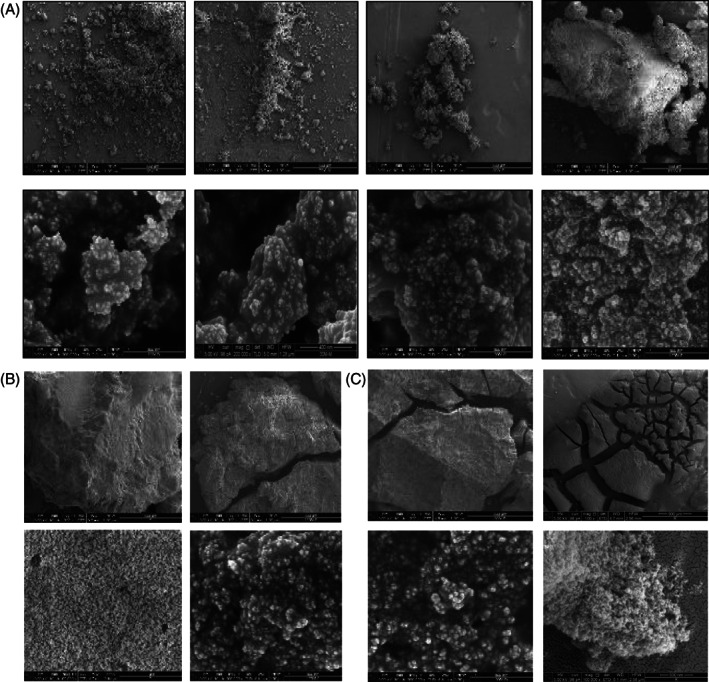
(A) SEM micrographs of the samples prepared with 1.0 wt % of *Centella asiatica* (*C. asiatica*) extract at different magnifications: 250× (upper line) and 250.000× (lower line). From left to right: S1, S2, S3 and S4 samples. (B) SEM micrographs of the samples prepared with 3.0 wt % of *C. asiatica* extract at different magnifications: 250× (upper line), 65.000× and 200.000× (lower line). From left to right: S5 and S6 samples. (C) SEM micrographs of the samples prepared with 5.0 wt % of *C. asiatica* extract at different magnifications: 250× and 100× (upper line), 200.000× and 100.000× (lower line). From left to right: S7 and S8 samples

SEM analysis revealed the presence of particle agglomeration, typical of the milled samples (Figure 2A–C). With an increment of the milling time, a decrease in the particle size and agglomeration are observed, simultaneously with the increase in the system disorder also observed in the XRPD patterns. The agglomeration is also favored by increasing the amount of natural extract probably due to the concurrent increase of the amount of propylene glycol contained in the extract.

**FIGURE 3:**
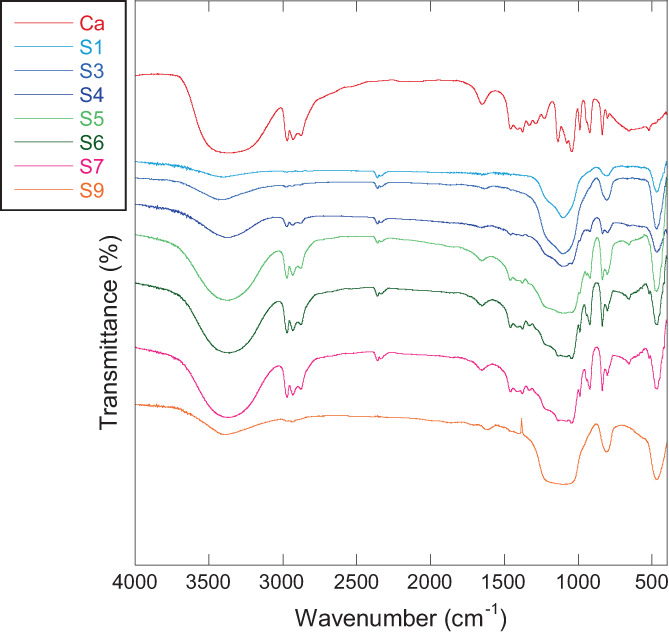
Fourier‐transform infrared spectroscopy (FTIR) spectra of the *Centella asiatica* (*C. asiatica*) glycolic extract (labeled as Ca) and of the *C. asiatica*‐SiO_2_ nanocomposites

FTIR analysis of all samples confirmed the presence of *C. asiatica* extract after the milling process at increasing milling time. The most representative FTIR spectra are reported in Figure 3. The signals of *C. asiatica* glycolic extract were more evident when the amount of extract was higher, except for the S9 sample. This difference could be related to the high amount of extract used to prepare this sample, which led to the production of a liquid product where the fumed silica is finally dispersed. It contrasts with the other samples, where the extract is dispersed in the silica matrix that protects it from any type of chemical degradation during the milling process.

**FIGURE 4:**
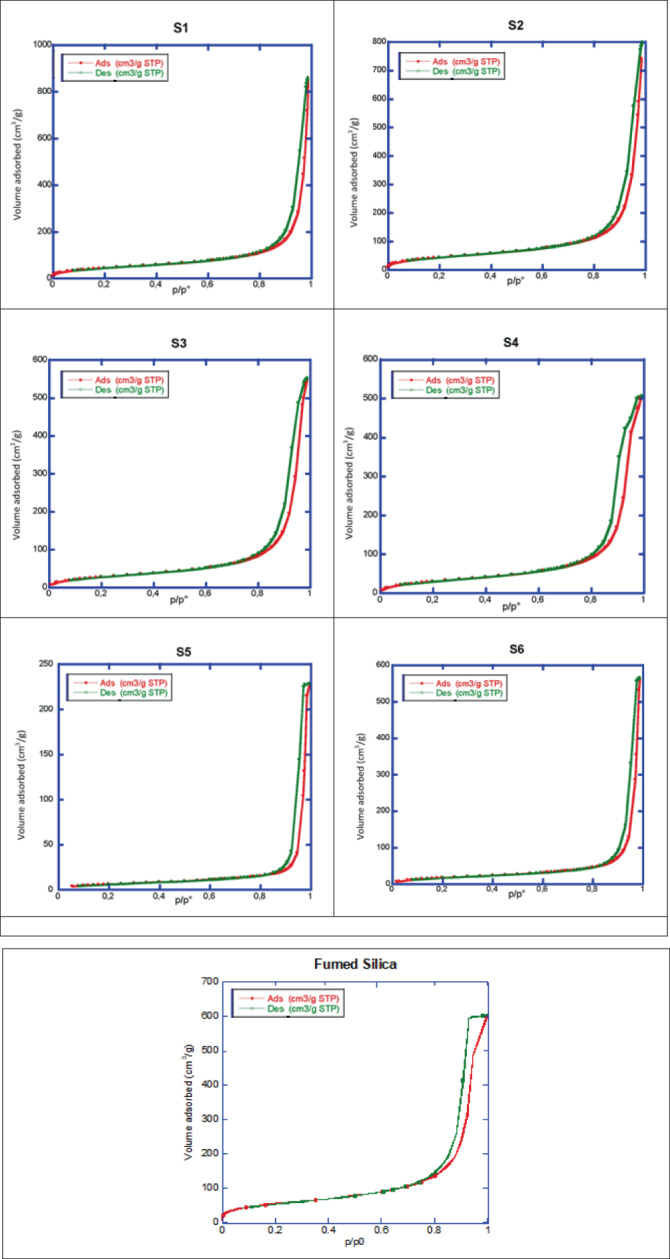
Nitrogen adsorption/desorption isotherms for the *Centella asiatica*‐SiO_2_ nanocomposites and the fumed silica at 77 K. Red and green curves refer to adsorption and desorption, respectively

All analyzed nanocomposites exhibited type IV isotherms with H1 hysteresis loop (Figure 4), typical of mesoporous materials. S7, S8 and S9 samples were not measurable because they were semisolid and sticky or liquid (S9). The SSA and porosity of the samples were measured (Table 1). The SSA of the nanocomposites was lower than that of the pure fumed silica. Moreover, when comparing the SSA values and the pore volumes (Vp) of the nanocomposites prepared with the lower amount of extract (1.0 wt %) a decrease is observed with increasing milling time. The observed behavior indicates that the milling process promotes the inclusion of the extract in the silica pores. Lower values of SSA and Vp are observed in the second series of samples, containing an extract amount equal to 3.0 wt %. This increase in the extract amount obviously induces the inclusion of a higher amount of extract within the silica pores as well as the silica particle agglomeration, which contribute to the rapid decrease of SSA and Vp values compared to samples containing 1.0 wt % of extract.

**FIGURE 5:**
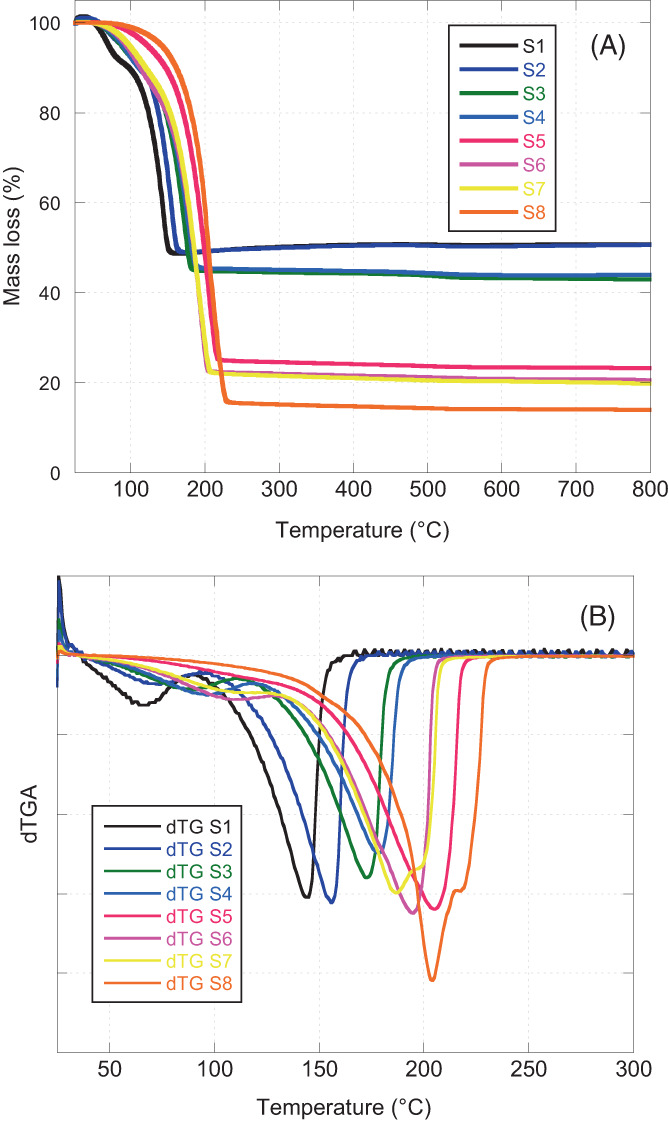
Thermogravimetric (TG, 5a) and derivative (dTG, 5b) curves of the *Centella asiatica*‐SiO_2_ nanocomposites

The presence of the natural extract in the nanocomposites was also confirmed by the thermogravimetric analysis (Figure 5A,B). The results indicate an increasing mass loss with the increment of the amount of the extract in the nanocomposites. A first mass loss is evident at around 100 °C, attributable to the evaporation of the water contained in the sample and the decomposition of the bioactive, while a second loss is observed in the range between 150 and 230 °C due to the elimination of the propylene glycol. The peaks of the derivative curves (dTG) also confirm the two mass losses (Figure 5B).

### 3.2 Radical scavenging activity of samples by DPPH test

**FIGURE 6:**
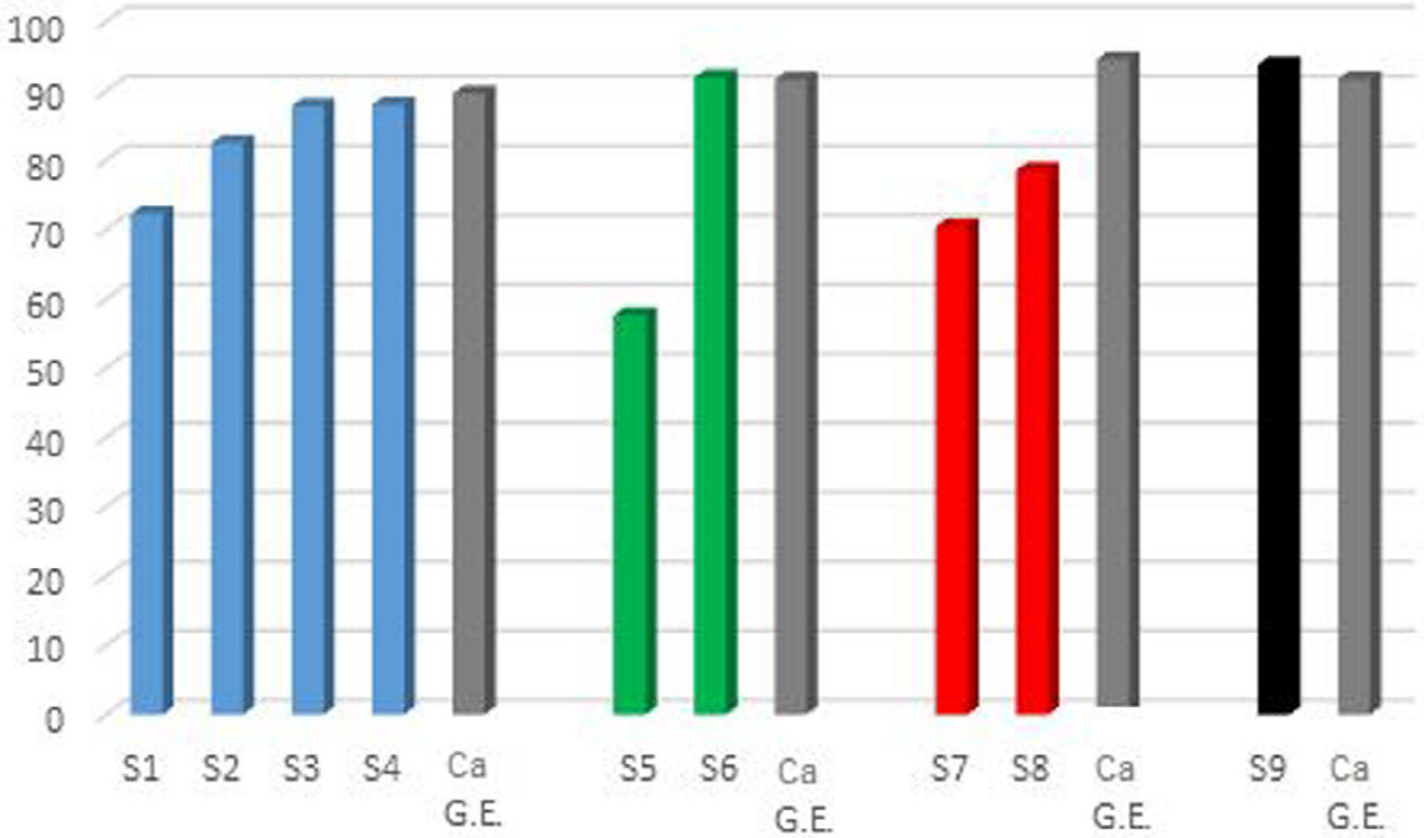
Antioxidant activity for the *Centella asiatica* (*C. asiatica*)‐SiO_2_ nanocomposites and for comparison pure *C. asiatica* glycolic extract (labeled as Ca G.E.)

Each series of the extract‐SiO_2_ nanocomposites (1.0, 3.0, 5.0, and 10.0 wt % of dry extract content) have an increased antioxidant activity with an increment of the milling time (Figure 6). It could be correlated to the improved inclusion of the extract in the silica pores, as observed by the nitrogen adsorption/desorption results, which could protect the extract from any physical and chemical degradation. The highest antioxidant activity values were observed for the nanocomposites prepared with the highest milling times (60 and 120 minutes), particularly evident with the increased content of the dry extract (i.e., 3 and 5 wt %). Furthermore, the antioxidant capacities of these samples are very similar to those of the pure extract solutions at the same concentrations (*C. asiatica* 1.0, 3.0, 5.0, and 10.0 wt %), confirming the capability of the silica to protect the extract without affecting its antioxidant power.

### 3.3 Biocompatibility and protective effect of the *C. asiatica* extract‐SiO_2_ nanocomposites

**FIGURE 7:**
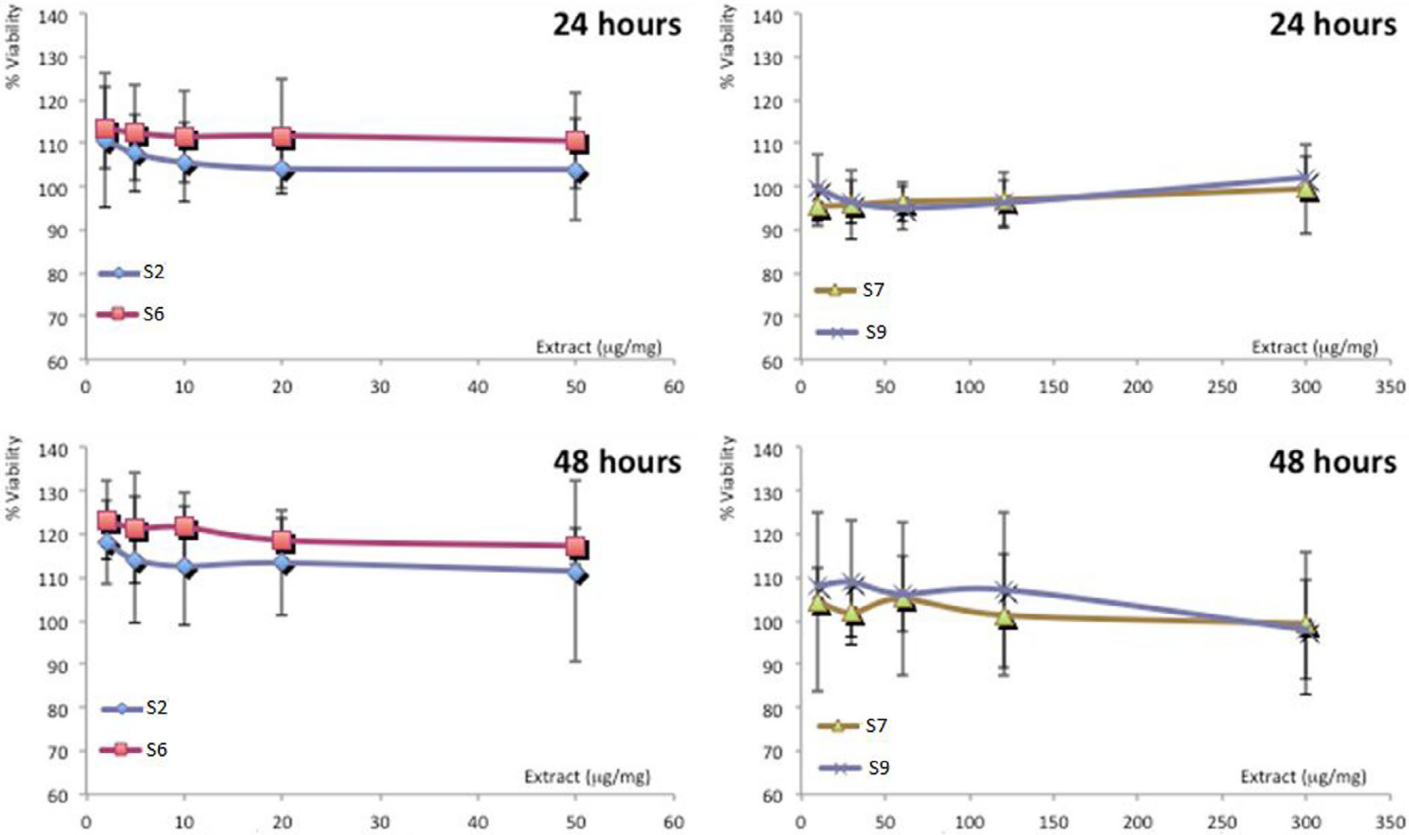
Viability of keratinocytes treated for 24 and 48 h with *Centella asiatica* extract containing nanocomposites at different concentrations. Mean values ± standard deviations (error bars) are reported (*n* = 8)

Human keratinocytes, as main cells of epidermis, were used to evaluate the biocompatibility of four representative samples. Cells were incubated with the formulations for 48 h and the cell viability was measured (Figure 7). Any cytotoxicity has been observed for the four samples, demonstrating the high biocompatibility of the formulations (Figure 7). In particular, S2 and S6 samples are able to promote keratinocyte proliferation, as the viability was always higher than 110% irrespective of the concentration used. The biocompatibility of S7 and S9 samples is also high (around 100%), but the promotion of the cell proliferation is evident only after 48 h of treatment and is slightly lower than that observed using S2 and S6 samples.

**FIGURE 8:**
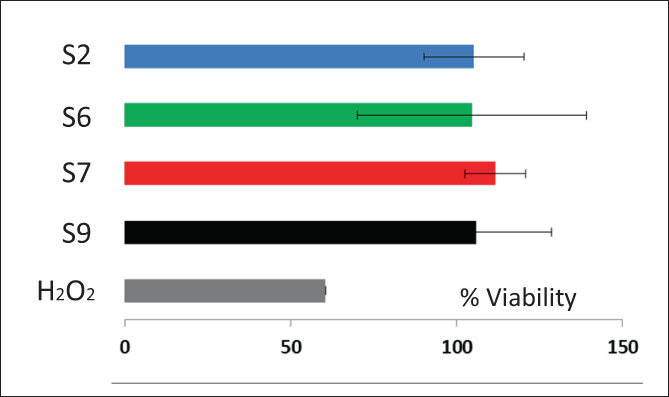
Viability values of keratinocytes stressed for 4 h with hydrogen peroxide and untreated or treated with *Centella asiatica* extract containing nanocomposites. Mean values ± standard deviations (error bars) are reported (*n* = 8)

The protective effect of the selected samples on cells damaged by hydrogen peroxide was also demonstrated. The stress of cells with hydrogen peroxide significantly reduces the cell viability, which was around 60% (Figure 8). On the contrary, the simultaneous treatment of stressed cells with the selected nanocomposites favors the complete restoring of the healthy conditions, reaching a cell viability always higher than 100%.

## 4 CONCLUSIONS

The use of biomaterials based on medicinal plants' constituents could provide a valuable strategy to improve the topical treatment of skin damages avoiding the use of systemic treatments. In particular, plant extracts are effective on the prevention and treatment of damages related to oxidative stress, which can favor the onset of pathological conditions or retard their healing.^30^ Although literature already reports on the benefits of medicinal plants, their use is somehow limited by the instability of the natural products to physical and chemical degradation as well as to metabolism after administration. Therefore, a decreased bioavailability of the active molecules is achieved, which results in less or no therapeutic.^31^, ^32^ Nanotechnology could represent a feasible solution to these limits, offering a wide selection of nano drug delivery systems for the incorporation of the natural products obtaining functional biomaterials. This aids to protect the active molecules from any type of degradation and increases their solubility in the body fluids, thus enhancing bioavailability and pharmacological activity.^32^, ^33^, ^34^, ^35^, ^36^

According to this purpose, the prepared novel silica‐based biomaterials containing *C. asiatica* glycolic extract, totally accomplish the extract protection, and controlled release as previously found in our studies.^18^ Ball milling is revealed a feasible technique for the preparation of *C. asiatica* extract‐SiO_2_ nanocomposites. The milling process promotes the inclusion of the *C. asiatica* glycolic extract within the silica pores without affecting the extract properties. High antioxidant activity, high biocompatibility, and protective effect on cells against hydrogen peroxide was observed for all nanocomposites, even for the samples with low content of *C. asiatica* glycolic extract. Given that, the obtained nanocomposite simultaneously could act as scavenger neutralizing and tissue regenerator creating the ideal condition to favor the skin repair.^21^, ^22^ Our synthetic approach is completely new and consists in the fine dispersion of drugs or natural extracts in a porous silica matrix by a simple ball milling one‐pot approach. The physico‐chemical characterization showed the incorporation of the natural extract in the silica pores promoted by the milling process, and the formation of the novel biomaterial in the form of a nanocomposite. This biomaterial retains the antioxidant activity of the *C. asiatica* extract after the milling process as demonstrated by the DPPH assay. High biocompatibility of the nanocomposites as well as cell protection capability against oxygen radicals were also demonstrated by the in‐vitro studies. These results further underlined the high biocompatibility of *C. asiatica* extract loaded in the nanocomposites along with their ability to promote the effectiveness of the extract in counteracting oxidative stress of cells and tissues. Along with 100% biocompatibility, high antioxidant activity, and cell protection from oxidative stress, our strategy also allows a fast and low‐cost preparation as well as easy up‐scaling of effective products.^24^, ^37^ In fact, while technologies are in principle available for large‐scale fabrication, production of biomaterials is still expensive because of high‐cost raw materials and equipment needed for their manufacturing. In addition, the production processes are usually complicated involving many steps.

Further studies on the preparation of topical formulations containing our nanocomposites are planned to evaluate the in‐vivo efficacy of the formulations. Thus, our novel *C. asiatica* ‐ silica nanocomposites can be proposed for the preparation of topical pharmaceutical preparations and wound dressing.

## CONFLICT OF INTEREST

There are no conflicts to declare.

## Data Availability

The data that supports the findings of this study are available in the article
